# *Bacillus velezensis* CL197: a zearalenone detoxifying strain isolated from wheat with potential to be used in animal production

**DOI:** 10.1007/s11259-024-10552-4

**Published:** 2024-09-24

**Authors:** Paloma Bianca Orso, Alberto Gonçalves Evangelista, Tiago de Melo Nazareth, Carlos Luz, Keliani Bordin, Giuseppe Meca, Fernando Bittencourt Luciano

**Affiliations:** 1 Graduate Program in Animal Science, School of Medicine and Life Sciences, Pontifícia Universidade Católica do Paraná, R. Imaculada Conceição, 1155 - Prado Velho , Curitiba, PR 80215-901 Brazil; 2 Polytechnic School, Pontifícia Universidade Católica do Paraná, R. Imaculada Conceição, 1155 - Prado Velho, Curitiba, PR 80215-901 Brazil; 3 Departament Medicina Preventiva i Salut Pública, Ciències de l’Alimentació, Toxicologia i Medicina Legal, Facultad de Farmàcia, Universitat de València, Av. de Vicent Andrés Estellés s/n, 46100 Burjassot, València, Spain

**Keywords:** Biocontrol, Mycotoxin, Bacterial screening, Food production chain, Agriculture

## Abstract

Zearalenone (ZEA) is a mycotoxin produced by *Fusarium* species, and cause contamination of food and feed, with impacts in animal production and in food production chain. Effective detoxifying methods, such as biodegradation, are therefore required. This study aimed to isolate microorganisms and screen ZEA detoxifying strains. As a result, 197 microorganisms were isolated, and six were initially selected after colorimetric screening. ZEA (1 µg/mL) was added to culture media, and after 24 h, all six microorganisms were able to degrade ZEA, without the formation of α-ZOL. One isolate eliminated ~ 99% of ZEA and was identified as *Bacillus velezensis* CL197. ZEA metabolites produced by the bacteria were evaluated, and no metabolites with greater or similar toxicity than ZEA were detected. This strain was applied to swine in vitro digestion, and up to 64% of ZEA was degraded. *B. velezensis* CL197 significantly degraded ZEA, demonstrating potential to be used as a detoxifying agent in the food production chain as a biocontrol agent.

## Introduction

Zearalenone (ZEA) is an oestrogenic mycotoxin produced by several *Fusarium* species (Miller 2016). Its oestrogenic effect is due to its molecular similarity to estradiol – a potent estrogen (Kowalska et al. 2018). The main ZEA derivatives are α-zearalenol (α-ZOL) and β-zearalenol (β-ZOL), which, respectively, have more and less affinity to estrogen receptors when compared to ZEA (Metzler et al. 2010). Other forms of ZEA are α-zearalanol (α-ZAL) and β-zearalanol (β-ZAL), which possess greater toxicity than the original mycotoxin (Ropejko and Twarużek 2021).

The ingestion of ZEA comes mainly from grains, frequently from maize, but it can also be found in other cereals such as wheat, barley, oat, and rice (Miller 2016). In mammals, this mycotoxin can be detrimental to the reproductive system. The oestrogenic effects of ZEA are more expressive in swine, as they metabolize ZEA to α-ZOL more easily than other animals, which increases the toxicity of the parent mycotoxin (European Food Safety Authority 2011). Controlling animal feed production is extremely important since the consumption of ZEA by animals can result in mycotoxin deposits in organs and tissues that resist food processing and can reach consumers (Thapa et al. 2021). Thus, controlling this mycotoxin in animal health is essential for avoiding possible implications in human health.

In humans, ZEA may cause premature puberty (Massart and Saggese 2010), infertility, endometrial cancer (Pajewska et al. 2018), prostate cancer (Kowalska et al. 2018) and mammary cancer (Kuciel-Lisieska et al. 2008). It is classified as non-carcinogenic by the lnternational Agency for Research on Cancer (1993). However, considering the date of the last classification and the discoveries made since then, the mycotoxin should be reclassified to reflect its public health significance and hazard.

Considering ZEA’s toxic effects, it is essential to reduce its contamination levels. Mycotoxin decontamination can be achieved by chemical (Nazareth et al. 2019; Evangelista et al. 2021a), physical (Avantaggiato et al. 2005; Nones et al. 2021) or biological methods. The latter, uses microorganisms or enzymes that degrade mycotoxins or adsorve them, reducing their toxicity, which has been proven to be a cost-effective measure (Tan et al. 2014; Shi et al. 2018; Ndiaye et al. 2022; Evangelista et al. 2024).

Several microorganisms can degrade ZEA, such as *Pseudomonas* (Tan et al. 2014), *Bacillus* (Tinyiro et al. 2011), *Lactobacillus* (Taheur et al. 2017; Adunphatcharaphon et al. 2021), and some fungi of the genus *Aspergillus* and *Rhizopus* (Brodehl et al. 2014).

Therefore, this study aimed to screen microorganisms from wheat and corn fields with ZEA biodegradation potential selecting them based on esterase activity. The study also aimed to identify the most promising strains and the decontamination byproducts. Finally, we evaluated the capacity of the microorganisms to metabolize ZEA during simulated swine digestion in vitro, searching for the application of potential microbial strains.

## Materials and methods

### Chemicals and culture media

Potato dextrose agar (PDA), brain heart infusion (BHI) agar, 1-naphthyl acetate (≥ 98%), fast blue B salt, pepsin (≥ 250 U/mg), ZEA and α-ZOL analytical standards, pancreatin (4 × USP), and bile salts were obtained from Sigma-Aldrich (St. Louis, MO, USA). High-pressure liquid chromatography (HPLC)-grade methanol and acetonitrile, and De Man, Rogosa, and Sharpe (MRS) agar, were purchased from Merck (Burlington, MA, USA). Liquid chromatography/mass spectrometry (LC/MS)-grade acetonitrile and formic acid were purchased from ThermoFisher Scientific (Waltham, MA, USA). Tryptone soy broth (TSB) and bacteriological peptone were obtained from HiMedia (Mumbai, India), and ethyl acetate and glycerol from Alphatec (Rio das Ostras, RJ, Brazil). Ultra-pure water (< 18 MΩ/cm resistivity) was obtained from a Milli-Q purification system (Merck Millipore, Burlington, Massachusetts, USA).

### Microorganisms isolation

Bacteria and yeast were isolated from corn and wheat fields – cereal and soil – located in *Campo Largo* (25° 27’ 32” S; 49° 31’ 40” W), *Agudos do Sul* (25° 59’ 34” S; 49° 20’ 06” W), and *Curitiba* (25° 25’ 47” S; 49° 16’ 19” W), cities from the Southern region of Brazil. For the isolation, 25 g of samples were diluted in 225 mL of 0.1% peptone water using a sterile bag. The samples were homogenized using a stomacher (IUL Instruments, Ciutat d’Asuncion, Barcelona, Spain) for 60s and submitted to serial dilution. Each dilution was plated on PDA, MRS agar, and BHI agar, and incubated at 30 °C for 48 h up to 5d. Colonies with different morphologies were cultivated onto new agar plates until complete isolation (Jalali et al. 2020). After isolation, the microorganisms were kept at TSB, MRS broth or BHI broth + 40% glycerol, at -80 °C, until further analysis. Before analysis, the isolates were cultured at least twice in the same culture media.

### Screening of microorganisms for ZEA biodegradation using esterase activity assay

Microorganisms with high esterase activities possess the potential to reduce ZEA contamination by degrading the mycotoxin macrocyclic lactone structure (Wang et al. 2017). Therefore, the esterase activity can be used as a screening test for ZEA biodegradation.

For the esterase activity assay, each isolate was grown in TSB for 24 h at 30 °C in a rotary shaker (Solab, Piracicaba, Brazil) set at 150 rpm. Thereafter, 100 µL of each isolate from a ~ 10^9^ CFU/mL inoculum (treatment), sterile distilled water (blank), or sterile broth culture media (control) were added to microtubes, in combination with the following preloaded reagents: 87 µL of fast blue B solution (2.5 mg/mL in distilled water) and 13 µL of 1-naphthyl acetate solution (2.5 mg/mL in ethanol). The tubes were incubated at 37 °C for 10 min and then centrifuged (Capp, Nordhausen, Germany) at 2000 × *g* for 10 min. The supernatant was placed into a 96-well microplate and the absorbance was observed in a plate reader (Molecular Devices, San Jose, CA, USA) at 524 nm. In the presence of esterase, colored complexes are formed, increasing absorbance values (Wang et al. 2017). Therefore, strains with absorbance values at least twice as high as control samples were selected.

### Zearalenone biodegradation assay

The selected isolates from the esterase test were grown TSB at 30 °C for 24 h on rotary shaker set at 150 rpm. Then, 1 mL from a ~ 10^9^ CFU/mL inoculum, was transferred to an microtube and 1 µg of ZEA was added. Tubes were incubated for 24 h at 30 °C in a rotary shaker at 150 rpm. The control group used sterile broth. After incubation, ZEA was extracted three times with 2 mL of ethyl acetate, with vigorous agitation and sonication (Unique, São Paulo, Brazil). The organic phases were combined, completely evaporated, resuspended in HPLC-grade methanol, and filtered through a 0.22 μm nylon filter. The samples were firstly analyzed for ZEA and α-ZOL content using HPLC (1220 Infinity, Agilent Technologies, Santa Clara, CA) with a fluorescence detector (HPLC-FLD; 1260 Infinity, Agilent Technologies, Santa Clara, CA) at excitation and emission wavelength of 236 nm. The stationary phase was a Gemini C18 column (Phenomenex, Torrance, CA), 4.6 mm x 150 mm, 110 Å, 3 μm particle size, and the isocratic mobile phase was ultrapure water: acetonitrile at the proportion of 60:40 and flow rate of 1 mL/min. Data were analyzed using Agilent OpenLab CDS ChemStation Editor (Agilent Technologies, Santa Clara, CA). Standard curves were produced using ZEA and α-ZOL concentrations varying from 0.02 to 1.2 µg/mL.

ZEA degradation rate (D_%_) was calculated according to the equation:

$$\:{D}_{\%}=(1-{(A}_{treatment}/{A}_{control}\left)\right)\times\:100$$

where A_treatment_ and A_control_ are the area obtained from the chromatogram related to ZEA content in the treatment and control samples.

The extraction recovery rate (%R) was calculated as follows:

$$\:\%R=\left({C}_{detected}/{C}_{added}\right)\times\:100$$

where C_detected_ is the calculated concentration from the standard curve, and C_added_ is the initially added concentration.

The detection (LOD) and quantification (LOQ) limits were determined as the ZEA or α-ZOL concentration corresponding to 3 and 10 times the noise area, respectively. The broth matrix effect was evaluated by comparing the angular coefficient from the standard curve diluted in solvent and broth. The strain with the most significant degradation rate of ZEA, but without the formation of α-ZOL was submitted to ZEA metabolites characterization, assessing possible degradation mechanisms, ZEA biodegradation kinetics, genetic identification, and in vitro swine digestion.

### Zearalenone metabolites characterization

The selected strain was again evaluated for ZEA degradation using 2 µg/mL of ZEA, as described in the “Zearalenone biodegradation assay” section. After ethyl acetate extraction, samples were analyzed using ultra-high pressure liquid chromatography quadrupole time-of-flight mass spectrometry (UHPLC-MS/qTOF) to determine ZEA degradation and the metabolites produced.

Chromatographic analysis was performed using an HPLC (1220 Infinity, Agilent, Santa Clara, CA, USA) equipped with an autosampler, vacuum degasser, and binary pump. Analyte separation was carried out on a Gemini C18 column, 50 mm × 2 mm, 110 Å, 3 μm particle size (Phenomenex, Torrance, CA, USA). The mobile phases were ultra-pure water + 0.1% formic acid (solvent A) and acetonitrile + 0.1% formic acid (solvent B), with a flow rate of 0.3 mL/min in a gradient (0 min, 5% B; 30 min, 95% B; 35 min, 5% B), and a run time of 35 min. The injection volume was 5 µL.

For mass spectrometry analyses, a MS/qTOF (6540 Agilent Ultra High-Definition Accurate Mass, Agilent Technologies, Santa Clara, CA), coupled to an Agilent Dual Jet Stream electrospray ionization (Dual AJS ESI, Agilent Technologies, Santa Clara, CA) interface operating in positive ion mode was used. Optimized mass spectrometry parameters included: fragment voltage of 175 V; capillary voltage of 3.5 kV; collision energy of 10, 20, and 40 eV, nebulizer pressure of 30 psi; drying gas flow (N_2_) of 8 L/min, and temperature of 350 °C. Data analysis was performed using MassHunter Qualitative Analysis Software B.08.00 (Agilent Technologies, Santa Clara, CA) (Escrivá et al. 2021).

### Genetic identification

DNA was extracted from approximately 50 mg of cells using the UltraClean Tissue and Cells DNA Extraction kit (MoBio, San Diego, CA). DNA purity was evaluated using NanoDrop2000 (ThermoFisher Scientific, Waltham, MA). DNA amplification was performed in a Thermal cycler (ThermoFisher Scientific, Waltham, MA) using 20 ng of DNA purified with GoTaq (Promega, São Paulo, Brazil). The 27 F/1492R primers were used for the 16 S region. The products were analyzed by 1% agarose gel electrophoresis and the reactions were enzymatically purified with ExoI/SAP (ThermoFisher Scientific, Waltham, MA), according to the manufacturer’s manual. PCR products were marked with BigDye v3.1 (ThermoFisher Scientific, Waltham, MA) in a reaction containing 50 ng of DNA and performed according to the manufacturer’s instructions. The marked products were precipitated with 20% ammonium acetate 7.5 M and three volumes of pure ethanol and resuspended in 10 µL of HiDi-formamide. The samples were sequenced in the Genetic Analyzer 3500xL (ThermoFisher Scientific, Waltham, MA), and data analysis was performed using the Sequencing Analysis v5.4 software. The sequences were trimmed with Phred score > 25 and were assembled in Cap3 program. The contig was compared with nr database (NCBI) using the BLASTn program.

### Analysis of ZEA detoxification mechanism

The identified strain, *Bacillus velezensis*, named CL197 and deposited in the Microorganism Collection of the Agri-Food Research and Innovation Laboratory at the Pontifical Catholic University of Paraná, was grown for 24 h on TSB at 30 °C and 150 rpm. Then, a sample of the fermented broth was autoclaved at 121 °C for 15 min, and another sample was centrifuged (Capp, Nordhausen, Germany) at 2000 × *g* for 10 min and filtered through a sterile 0.22 μm PTFE hydrophilic syringe filter. The resulting fractions were then subjected to the ZEA biodegradation assay previously described, analyzed by HPLC-FLD.

### ZEA biodegradation kinetics

The strain was incubated on TSB broth with 1 µg ZEA/mL on rotary shaker (150 rpm). Samples were then extracted at 0, 1, 3, 6, 12 and 24 h and analyzed for ZEA and α-ZOL under HPLC-FLD. ZEA degradation was considered as first order kinetic model, and was calculated using the equation:

$$\:{C}_{t}={C}_{0}\times\:{e}^{-kt}$$

where C_t_ is the ZEA concentration at the respective time (t) and C_0_ is the initial concentration (Wang et al. 2017).

The time to degrade 50% of the initial concentration (t_1/2_) was calculated with the following equation:

$$\:{t}_{1/2}=\text{ln}\;(2/k)$$

### ZEA biodegradation in simulated swine digestion

The simulated swine digestion was done according to Evangelista et al. (2021b), with few modifications. For the gastric phase, tubes containing 2 mL of water contaminated with 0.5 µg/mL of ZEA were added 20 mL of phosphate buffer pH 6.0, 50 U of pepsin, and 1 mL of the cultured TSB broth (10^8^ CFU/mL) or sterile TSB. The pH was adjusted to 2.0 ± 0.2 with hydrochloric acid 0.2 M. The tubes were incubated for 2 h at 39 °C in a Dubnoff orbital shaking water bath (Tecnal, Piracicaba, San Pablo, Brazil) with agitation set at 250 rpm.

For the intestinal phase, 7 mL of phosphate buffer pH 6.8 and 100 mg of pancreatin were added to the gastric phase tubes. The pH was adjusted to 6.8 ± 0.2 with sodium hydroxide 0.6 M. Tubes were then incubated for 4 h under the same conditions as the previous phase.

Additionally, the effect of adding the strain only in the intestinal phase was also evaluated. The insertion of bacteria only in the intestinal phase simulates an already colonized intestine, while adding bacteria from the beginning of the digestion simulates the constant supply via feed or drinking water.

After the initial, gastric, and intestinal phases, the tubes were extracted for the detection of ZEA and α-ZOL using HPLC-FLD. The matrix effect of the digestion liquids was also determined.

Samples of each step (before the gastric phase, gastric phase, and intestinal phase) were plated on PDA agar to evaluate the survival rate of the bacterium throughout the digestion. The plates were incubated at 30 °C for 24 h and the colonies with the morphology of the selected strain were counted.

### Statistical analysis

All analyzes were performed at least twice in triplicate. The results are presented as mean ± standard deviation. Statistical analysis was performed using GraphPad Prism 7.0 software (Boston, MA). The results were subjected to the D’Agostino-Pearson normality test. The Mann-Whitney test was used to analyze esterase activity screening and the difference in ZEA degradation between control and treatment in culture media. For the simulated swine digestion, the Mann-Whitney test was used to analyze the difference in control and treatment at the same time, and the Kurskall-Wallis test followed by Dunn’s multiple comparison test was used to analyze the difference in control and treatment among different times. The significance level was *p* < 0.05.

## Results and discussion

### Microorganisms isolation and esterase activity-based selection

One hundred and ninety-seven microorganisms were isolated from the collected samples, including bacteria and yeast. Of these isolates, 20.81% showed esterase activity. Six isolates met the established methodological parameters (absorbance values at least twice as high as control) and were selected for ZEA biodegradation (Table 1).

**Table 1:** Absorbance values of selected strains in esterase activity screening. Higher absorbance represents increased esterase activity by transforming 1-naphthyl acetate into 1-naphthol, which reacts with the fast Blue B dye. Absorbance was measured at 524 nm

Sample	Absorbance
Control	0.580 ± 0.128
Isolate 62	1.437 ± 0.324
Isolate 123	1.883 ± 0.543
Isolate 162	1.367 ± 0.107
Isolate 140	1.282 ± 0.213
Isolate 178	1.268 ± 0.038
Isolate 197	1.290 ± 0.187

### HPLC-FLD methodology validation and microbial reduction of ZEA

The extraction methodology presented a recovery rate of 98.5 ± 0.21%, validating the extraction process, and the culture media had no matrix effect. For ZEA and α-ZOL, the LOD were 4 and 20 ng/mL, and the LOQ were 10 and 65 ng/mL, respectively.

**Fig. 1:**
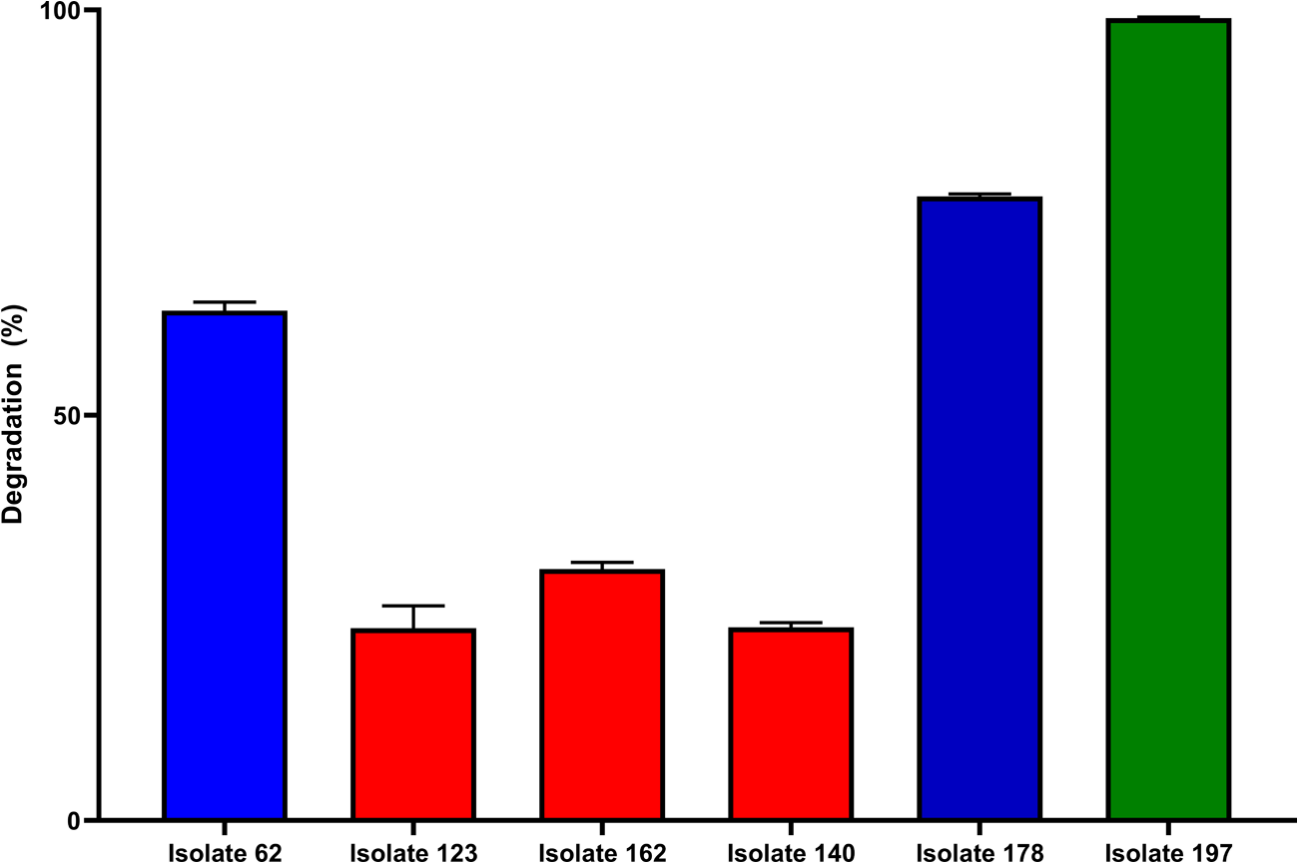
Zearalenone (1 µg/mL) degradation rate by esterase-selected microbial strains

The esterase assay was an effective screening method, as all the selected strains were able to significantly reduce the initial concentration of 1 µg/mL of ZEA after 24 h. Out of the six strains, three presented more prominent reduction rates of 62.9 ± 1.03%, 77.0 ± 0.29%, and 99.0 ± 0.11%, while the other three degraded ~ 20–30% of ZEA (Fig. 1).

No α-ZOL – the more oestrogenic ZEA metabolite – was detected in all samples, supporting the hypothesis that esterase enzymes may break the lactone ring and avoid the formation of more toxic compounds (Chen et al. 2018). The isolate 197 presented the best biodegradation activity and was selected for further steps.

### Characterization of the selected strain

The 16 S rRNA sequencing and analysis led to a high confidence (E < 1 × 10^−73^) and high identification percentage (99%) of similarity with *Bacillus velezensis*. The strain was then named *B. velezensis* CL197.

In UHPLC-MS/qTOF analyses, the ethyl acetate extraction process showed a recovery rate of 83.1 ± 1.31%, thus validating the extraction procedure. *B. velezensis* CL197 eliminated 96.3 ± 0.46% of ZEA, and several metabolites and ZEA conjugates were detected (Table 2). These compounds were detected in very low level, ranging from 0.010 to 0.092 µg/mL and some of the conjugates contained a glucose and/or sulfate moieties.

**Table 2:** Characterization of zearalenone (ZEA) byproducts formed after *Bacillus velezensis* CL197 metabolization. Identification was performed using ultra-high pressure liquid chromatography quadrupole time-of-flight mass spectrometry (UHPLC-MS/qTOF). Initial concentration of ZEA was 2 µg/mL

Metabolite molecular formula	Concentration¹	*m/z*	Presumptive compound	Reference
C6 H10 O7	0.012	193.0354	Glucuronic acid	Llorens et al. 2022
C32 H46 O16	0.071	685.2760	Zearalenol-Ac-diGlc	Righetti et al. 2018
C24 H34 O10	0.043	527.2126	Zearalenol-Glc	Panel and Chain 2016
C24 H32 O10	0.010	525.1959	Zearalenone-4-beta-D-glucopyranoside	Schneweis et al. 2002
C30 H42 O15	0.070	641.2451	Zearalenone-di-Glc	Panel and Chain 2016
C27 H34 O13	0.092	611.1982	Zearalenone-MalGlc	Pfleger and Schwake-Anduschus 2023
C18 H26 O5	0.013	381.1916	Zeranol	Takemura et al. 2007
C18 H24 O8 S	0.023	459.1307	α- or β-zearalenol-Sulf	Panel and Chain 2016

¹Equivalent concentration in µg/mL of ZEA

*Glc* glucoside, *Ac* acetyl, *Mal* malonyl, *Sulf* sulfate

As previously mentioned, α-ZOL is the more oestrogenic ZEA metabolite (Chen et al. 2018), and it is extremely important that it is not formed from ZEA degradation. Although detected in the control group, it was observed that the bacterium was able to eliminate the compound, and only 0.023 µg/mL of α-ZOL or β-ZOL was detected conjugated with sulfate. Zeranol (ZOL), also a more oestrogenic compound than ZEA (Takemura et al. 2007), was detected at a very low level (0.013 µg/mL) in comparison with the initial level of the parent mycotoxin.

*B. velezensis* is a gram-positive bacterium that forms biofilms (Dunlap et al. 2016). This bacterium can indirectly provide bioprotective effects to plants by stimulating systemic resistance (Fan et al. 2018), and directly by forming antifungal compounds and outcompeting fungal growth (Palazzini et al. 2016; Pandin et al. 2018; Luo et al. 2019). Studies of the secondary metabolite gene clusters of *B. velezensis* have identified many antibiotic compounds, such as bacilysin, surfactin, bacillaene, iturin, and bacillibactin (Palazzini et al. 2016; Diabankana et al. 2022). Furthermore, this species is associated with a ZEA-degrading enzyme called laccase (Li et al. 2020; Wang et al. 2021). Additionally, this bacterium can be used as a substitute for synthetic fertilizers and pesticides and to degrade toxic industrial by-products (Adeniji et al. 2019).

Several studies have demonstrated the ZEA degradation potential of *B. velezensis*; for instance, Guo et al. (2020) achieved 95% of ZEA degradation in culture media after 48 h of incubation with *B. velezensis*. Our results, along with those found in scientific databases, suggest that this strain could be an option for ZEA detoxification, with potential applications in agriculture and the food production chain.

### Probable ZEA detoxification mechanism and biodegradation kinetics

Mycotoxin detoxification can occur by adsorption of the mycotoxin on the bacterial cell wall or by degradation of its molecular structure. When *B. velezensis* CL197 was autoclaved, it was unable to reduce ZEA levels, indicating that the mechanism of mycotoxin reduction was most likely not related to cell wall adsorption since cell wall fragments were still present in the medium. After the fermented medium was filtered and became cell-free, the degradation of ZEA occurred at a lower rate of 73.8 ± 1.64%, confirming a predominantly enzymatic degradation rather than cellular adhesion of the mycotoxin. The lower degradation rate after filtering might be caused by the absence of bacterial biofilm, by the lack of intracellular enzymes, or by the absence of constant production of extracellular enzymes during the incubation period.

Microorganisms organized in biofilms have been shown to have increased enzyme production due to altered gene expression (Pandin et al. 2017), and when the biofilm is not present, enzyme availability may be reduced. A similar conclusion was reached by Xu et al. (2016), who reported little ZEA cellular adhesion (6.1%) by *B. amyloliquefaciens* ZDS-1 – a *B. velezensis* later heterotypic synonym – pointing enzymatic degradation as the major mechanism for ZEA removal. The authors also reported similar detoxifying rates as the present study, with 95.7% (1 µg ZEA/mL of original concentration) degradation of ZEA.

Several *Bacillus* and phylogenetically close genera are reported to enzymatically degrade ZEA in liquid media. *Lysinibacillus* ZJ-2016-1 completely removed 32 µg/mL of ZEA after 48 h of incubation, probably by an enzymatic reaction, without cell binding or absorption (Wang et al. 2018a). *Bacillus pumilus* ES-21 degraded 95.7% of 17.9 µg/mL ZEA within 24 h, an effect mainly attributed to enzymatic degradation rather than physical adsorption, according to the authors’ results (Wang et al. 2017). It is important to emphasize that the authors pointed out a reduction in ZEA levels without the detection of metabolites with greater toxicity, such as α-ZOL.

*B. velezensis* A2 completely degraded 7.45 µg/mL of ZEA in 72 h in Luria Bertani medium, and a daily dose of 0.2 mL of this broth containing the bacterium prevented ZEA-induced kidney damage in mice after 28 days (Wang et al. 2018b). The biodegradation capacity of this strain may be related to laccase production, as this protein can hydrolyze ZEA’s lactone ring (Wang et al. 2021). Additionally, strain A2 was able to completely eliminate 60 µg/kg of ZEA in mice feed after 5 days of fermentation, and daily ingestion of 6 g of the feed showed no pathological changes in mice liver after 28 days (Wang et al. 2018c).

**Fig. 2:**
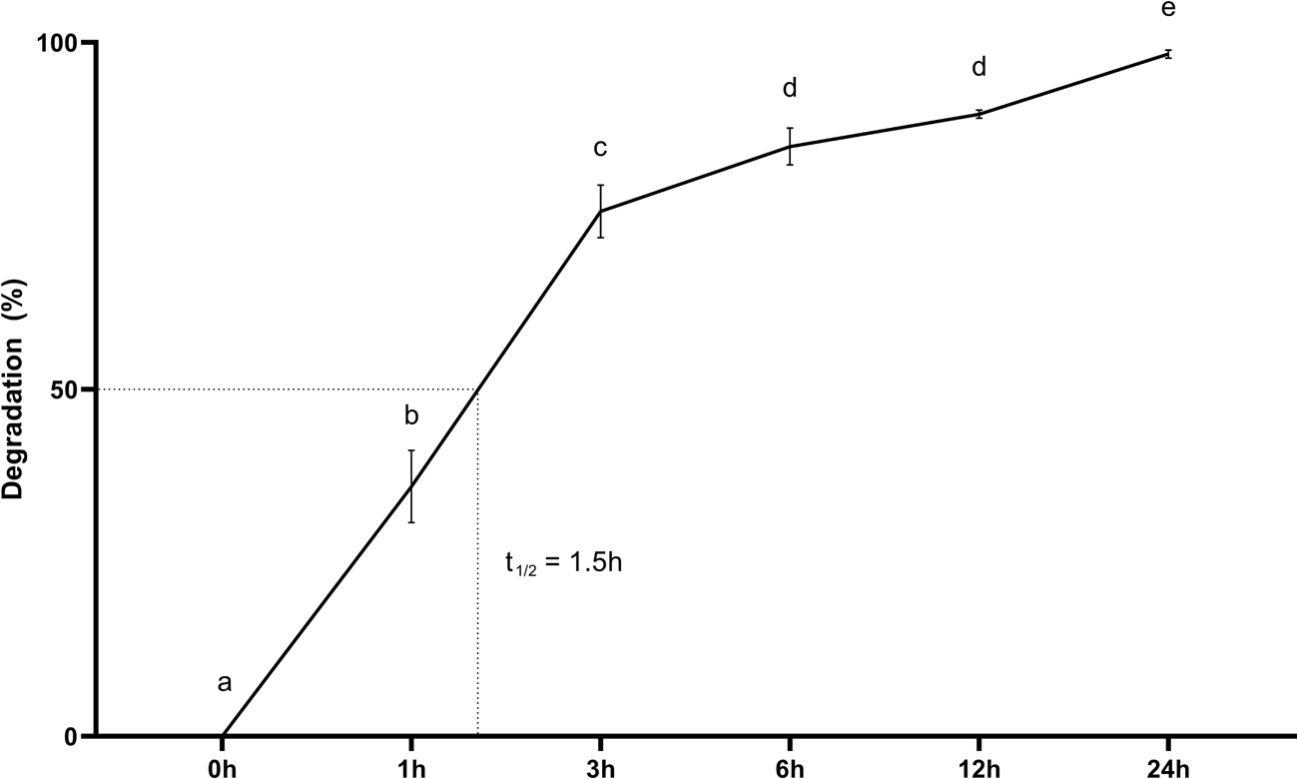
Kinetic curve of zearalenone (1 µg/mL) degradation by *Bacillus velezensis* CL197 at 30 °C for 24 h. Different lowercase letters indicate statistical difference (*p* < 0.05)

*B. velezensis* CL197 rapidly degraded ZEA. In three hours, almost 80% of ZEA was eliminated. The time to reduce 50% of the mycotoxin (t_1/2_) was calculated as 1.5 h (Fig. 2). This initial rapid degradation rate followed by the slower reaction as the experiment concludes is typical of enzymatic reactions (Penner 2001).

Enzymatic degradation is a more desirable result, since a simple adsorption, for example, makes the presence of the toxin undetectable initially, but later it can be released in the gastrointestinal tract to manifest its toxic effects. Thus, our results are in line with previous findings, and they support the possible use of *B. velezensis* CL197 as an efficient method to detoxify ZEA without the formation of metabolites with greater toxicity.

### Simulated swine digestion

**Fig. 3:**
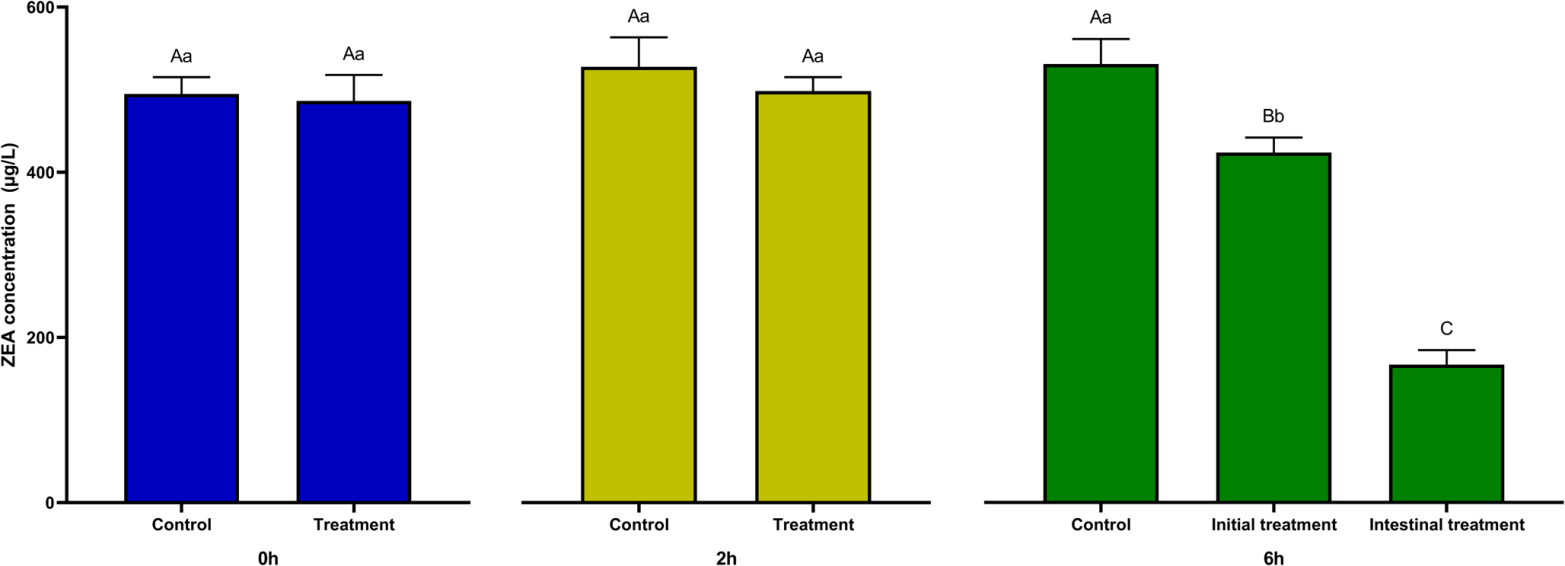
Zearalenone (0.5 µg/mL) degradation during swine simulated digestion. Control samples did not receive any microbial inoculum and treated samples were added with 10^8^ CFU/mL of *Bacillus velezensis* CL197. Light green bars represent the end of the gastric phase and dark green bars represent the end of intestinal phase. During intestinal phase, *B. velezensis* CL197 was originated from the gastric phase (initial treatment) or was introduced directly to the simulated intestinal digesta (intestinal treatment). Different lowercase letters in the same treatment at different times indicate statistical difference (*p* < 0.05). Different uppercase letters in different treatments at the same time indicate statistical difference (*p* < 0.05)

The simulated swine digestion evaluated the potential application of *B. velezensis* CL197 in feed as a ZEA detoxifying agent. After the gastric phase, no significant degradation was observed. However, after the intestinal phase, this bacterium was able to reduce ZEA levels. The addition of the strain only in the intestinal phase resulted in a significantly higher degradation rate (64.3 ± 0.03%) compared to when the microorganism passed through all digestion phases (18.9 ± 0.04%) (Fig. 3).

This lower degradation rate when passing through the stomach may be associated with the low pH, as it is not optimal for esterase enzymes (Lisboa et al. 2013), and the microorganism may be injured when exposed to acidic conditions (Ruiz-García et al. 2005), which can hinder its further activity. The degradation rate found in a strong acid environment (pH ≤ 4.0) was in line with previous studies with *Bacillus* strains; Xu et al. (2016) demonstrated that *B. amyloliquefaciens* ZDS-1 has greater potential to degrade ZEA in pH 6 and 7 (~ 95% degradation), followed by pH 8 (~ 60% degradation), pH 5 (~ 40% degradation), and pH 4, 9, and 10 (~ 5% degradation).

By the action of degradation primarily in the intestinal portion, it is presumed that the bacterium can prevent or reduce the levels of absorption of ZEA in the organ, leading to a decrease in its systemic effects. However, other factors must be considered; the adhesion of *B. velezensis* CL197 in intestinal cells is one of the most important factors, requiring further studies in vitro to verify the capacity of this strain to adhere on the surface of enterocytes.

**Fig. 4:**
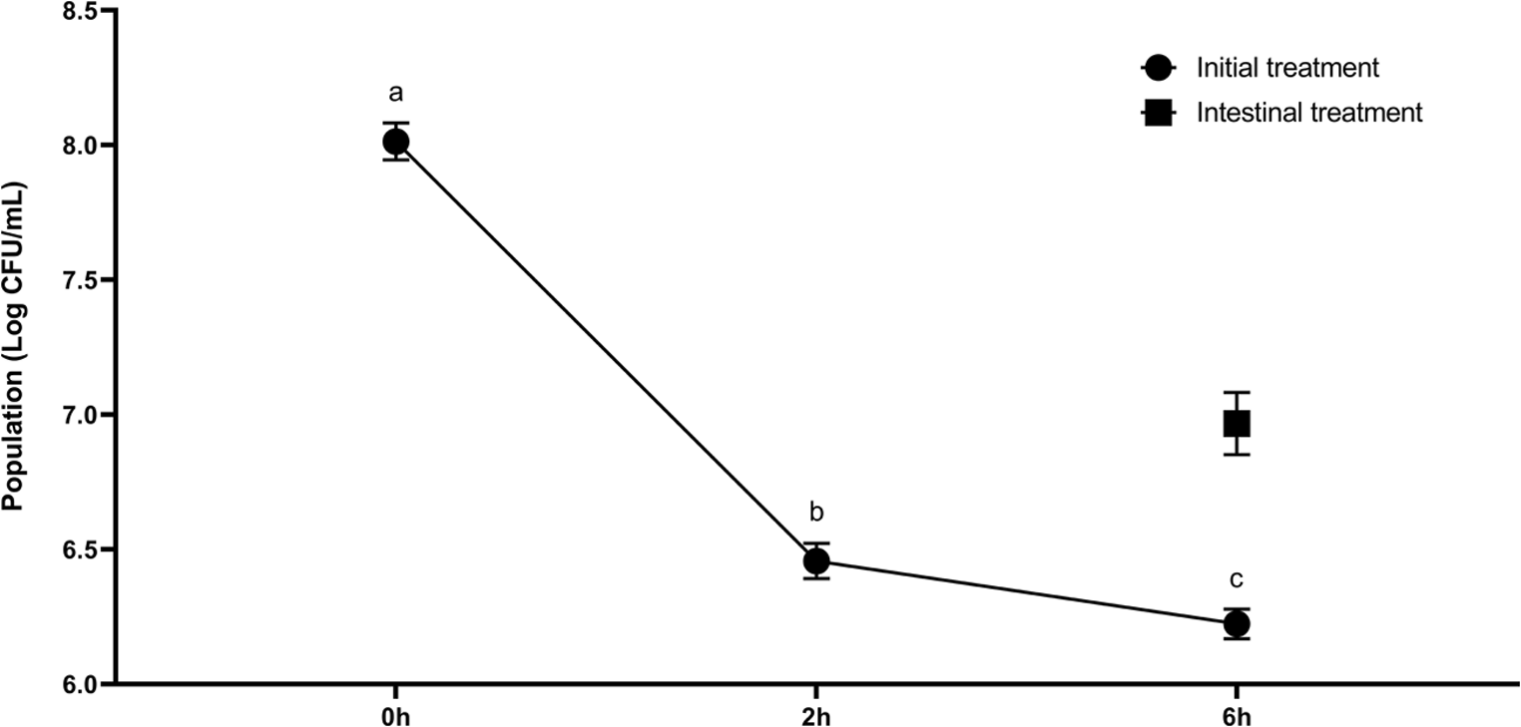
*Bacillus velezensis* CL197 population throughout digestion phases, expressed in log CFU/mL. Different lowercase letters indicate statistical difference (*p* < 0.05)

In terms of the survival rate of the bacterium, the reduction of 1.80 log CFU/mL on *B. velezensis* CL197 population after passing through the digestive system can be attributed to the low pH in the stomach. The microbial count reduction was less pronounced when *B. velezensis* was added only in the intestinal phase as the pH is higher in this environment (Fig. 4). Constant feeding of animals with this bacterium as an ingredient in animal feed or water may warrant sufficient population in the intestine to avoid the toxic effects of ZEA.

Ruiz-García et al. (2005) reported that *B. velezensis* 502 and CR-14b grow in a pH range from 5 to 10, indicating that strong acid environment is not favorable for *B. velezensis’* growth. Strain adaptation to a lower pH — done by exposition to an acid environment before passing the gastric phase (Cotter and Hill 2003) — or microencapsulation with release of cells in the intestinal tract might be effective to decrease the mortality rate throughout the digestion (Shori 2017) and improve ZEA degradation.

Even though the microbial count significantly decreased, the strain could survive to a significant level, reaching 6.2 ± 0.05 log CFU/mL at the end of the digestion. This survival rate indicates its potential to be used as a probiotic in animal feed, and it is in accordance with findings reported by Khalid et al. (2021), which suggests to *B. velezensis* as an emerging probiotic for animal feed.

The data involving *B. velezensis* in simulated swine digestion against mycotoxins are scarce, highlighting the importance of the present findings. Studies demonstrating reduction of ZEA levels by other microrganisms used in swine production were found. An in vivo study evaluated the effect of the addition of *B. subtilis* ANSB01G and *Devosia* ANSB714 against ZEA and deoxynivalenol (DON) in female gilts. The authors found that gilts fed with these bacterial strains displayed reduced harmful effects in the reproductive system (Shi et al. 2018).

Another research evaluated the protective effect of cell-free supernatant from a pool of probiotics combined with enzymes produced by *Aspergillus oryzae* on swine jejunal epithelial cells against the toxic effects of ZEA and aflatoxin B_1_ (Huang et al. 2019). These studies tested mycotoxin co-occurrence, which can lead to synergistic toxic effects (Corrêa et al. 2018). The authors found protective effects as cytotoxicity was reduced by the addition of the tested compounds.

These findings demonstrate the potential application of detoxifying bacteria or enzymes in feed, with the aim of promoting better mycotoxin protection throughout the food production chain, thereby increasing food safety and quality, and ultimately improving human health. Although no comprehensive data on ZEA contamination in meat worldwide were found, regional data from Jordan indicate a prevalence of up to 30% in samples of animal meat and edible organs, particularly kidneys and liver. This does not exclude the possibility of much greater contamination, as there is an underestimation of the mycotoxins in meat and meat products around the world (Kępińska-Pacelik and Biel 2021).

## Conclusion

The bacterium isolated in this research, *B. velezensis* CL197, showed great potential for use in the biodegradation of ZEA without the formation of compounds with greater toxicity. Its action likely occurs through an enzymatic route rather than adsorption, which is desirable, since the adsorption can be reversed, leading to toxic effects. The bacterium’s action in the culture medium was fast, reducing half of the toxin concentration in 1.5 h. The levels of toxin degraded are much higher than those commonly found in grains and animal feed. Furthermore, its effect on simulated swine digestion was adequate, reducing the concentration of ZEA in the intestinal phase, where it would be absorbed and lead to deleterious effects.

In order to harness the full potential of this bacterium within the food production industry, it is imperative to conduct comprehensive in vitro assessments. These analyses should primarily focus on exploring encapsulation techniques that can enhance the bacterium’s survivability during gastrointestinal transit. Furthermore, conducting in vivo investigations is crucial to gauge the bacterium’s efficacy within the host organism, while also evaluating its potential impact on food productivity. Thus, a multifaceted approach encompassing both in vitro and in vivo analyses is essential to unlock the bacterium’s applicative prowess in the food production chain.

## Data Availability

Data is provided within the manuscript.

## Abbreviations

ZEA: Zearalenona
α-ZOL: Alfa-zearalenol
β-ZOL: Beta-zearalenol
α-ZAL: Alfa-zearalanol
β-ZAL: Beta-zearalanol
PDA: Potato Dextrose Agar
BHI: Brain Heart Infusion
HPLC: High-Pressure Liquid Chromatography
LC/MS: Liquid Chromatography/Mass Spectrometry
LOD: Limit of Detection
LOQ: Limit of Quantification
UHPLC-MS/qTOF: Ultra-High Pressure Liquid Chromatography Quadrupole Time-of-Flight Mass Spectrometry
HPLC-FLD: High-Pressure Liquid Chromatography with Fluorescence Detection
MRS: De Man, Rogosa, and Sharpe
TSB: Tryptone Soy Broth
DON: Deoxynivalenol
D%: Degradation rate
%R: Recovery rate
